# Hand hygiene strategies

**DOI:** 10.3402/jchimp.v1i2.7200

**Published:** 2011-07-18

**Authors:** Eskandar Alex Yazaji

**Affiliations:** Department of Medicine, Union Memorial Hospital, Baltimore, Maryland

**Keywords:** hand hygiene, healthcare associated infections, multidisciplinary program, system change, accountability, education, feedback

## Abstract

Hand hygiene is one of the major players in preventing healthcare associated infections. However, healthcare workers compliance with hand hygiene continues to be a challenge. This article will address strategies to help improving hand hygiene compliance.

Patient safety is one of the most targeted areas in our evolving health care system, and hospital-acquired infections are a major threat to patient safety. Hand hygiene is an example of a simple precaution that can reduce hospital-acquired infections and improve patient safety.

According to the Centers for Disease Control and Prevention (CDC), pathogens in hospitals are commonly transmitted by hands. Adherence to hand hygiene is one of the most effective measures to prevent health care associated infections.

A decade ago, the rate of hand hygiene compliance in most US hospitals was often below 20% (1). Currently, hand hygiene compliance rates range between 30 and 70% for US hospitals; few have achieved and sustained compliance over 80% (1).

The Joint Commission recommendations regarding hand hygiene are as follows:

1. Wash with soap and water when:
  - Hands are visibly dirty or soiled with blood or other body fluids.
  - In case of contact with spore-forming pathogens such as *Clostridium difficile*.
2. Use of alcohol-based hand rubs is preferred for all other clinical situations.

The World Health Organization (WHO) identified the ‘five moments’ to use hand hygiene as follows:

1. Before patient contact,
2. After patient contact,
3. After contact with body fluids,
4. After contact with surfaces and objects in the immediate vicinity of the patient,
5. Before aseptic procedures.

The WHO also provides an excellent comprehensive hand hygiene improvement program (2). The components of this program are as follows:

1. System changes,
2. Training and education,
3. Evaluation and feedback,
4. Reminders in the workplace,
5. Institutional safety climate.

## System changes

It is very important to make sure that hand rubs are available inside and outside all patient rooms. Surveying health care workers regarding their acceptance of the alcohol hand rub products is essential to compliance with using those products. Another important factor is to ensure that dispensers are refilled and maintained properly.

## Training and education

All health care workers should be aware of the appropriate indications of hand hygiene (the five moments). Hospitals should invest in educating trainers, observers, and health care workers. Examples of education initiatives are lectures, computerized modules, training videos from the CDC and WHO, guidelines, and a hand hygiene week campaign.

## Evaluation and feedback

Hand hygiene compliance should be measured regularly and feedback must be given in a timely manner. Compliance can be measured by observation or by measuring soap and hand rub consumption. Observation by trained observers is currently the preferred method. Observers may use paper-based or electronic methods. It is preferred that the observer be unknown and not on his or her own unit to minimize conflict of interest.

Health care workers should receive periodic feedback, either positive or negative, regarding their compliance. Compliance rates should be reported by nursing unit and job description. Posting compliance rates on nursing units is very effective in fostering competition and identifying less compliant units or job category. Areas that need improvement must receive education and in-service sessions.

Some hospitals provide real-time feedback when hand hygiene is not performed. This can be a very effective tool to identify offenders and educate them quickly.

## Reminders in the workplace

Posters should be used widely in the health care setting emphasizing the indications for hand hygiene, such as the five moments, and the appropriate way to use hand rubs and soap. Posters should be changed regularly because they become less effective as health care workers become used to them. Hand hygiene screen savers, pens, and coffee mugs with reminders may also be used.

## Institutional safety climate

The medical board should make hand hygiene an institutional priority and provide the needed resources. Hand hygiene compliance rates should be reported regularly at high-level administrative meetings, such as nursing manager, medical executive, departmental, and performance improvement meetings. Senior clinicians and administrators should provide visible support for hand hygiene as a role model. Policies and procedures should be implemented to hold health care workers accountable for hand hygiene compliance.

Accountability has been challenging when it comes to physicians, mostly because physicians usually are not employed by hospitals. Hospitals have been reluctant to punish them for fear of alienating them and losing the business they bring in.

Low hand hygiene compliance rates are generally not a system problem anymore. They are now largely an accountability problem (1). As long as transgression carries no risk, some providers will ignore the rules because they believe that they are not at risk, they are too busy, or they do not believe hand hygiene is effective (1).

Other high-risk industries such as the airline industry use strict safety measures. For example, a pilot would not be allowed to fly without using the checklist. The health care industry should apply stricter safety and accountability measures as well. However, expecting strict adherence to safety standards before addressing the system is a mistake. There should be no punishment unless non-compliance is deliberate despite education, counseling, and system improvements. In case of health care worker chronic non-compliance despite feedback and education, an example of consequences might include a 1-week suspension and 2 hours of online education on infection prevention (1).

**Fig. 1:**
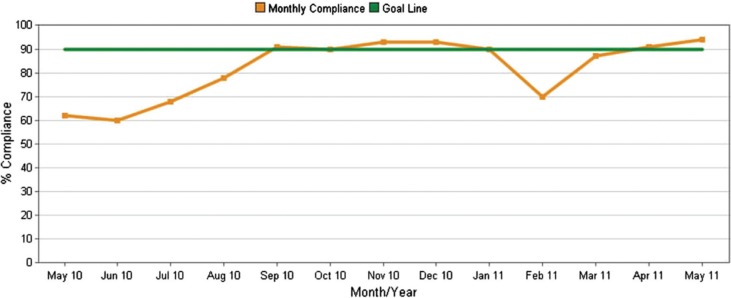
Hand hygiene compliance rate since May 2010. The hand hygiene campaign started in July 2010. The compliance rate improved significantly from around 60%–70% before the campaign to over 90% in most months thereafter.

At our institution, we implemented a hand hygiene campaign in July 2010 using the guidelines of the CDC, Joint Commission, and WHO. Our compliance rate with hand hygiene improved significantly from 60 to 70% in early 2010 to over 90% starting in September 2010 (Fig. 1).

In conclusion, performing hand hygiene before and after patient contact is one of the most important measures to prevent transmission of pathogens. A multidisciplinary program is necessary to achieve and sustain hand hygiene compliance. Vocal and visible support of senior clinicians and high-level administrators is crucial for the success of hand hygiene program. Besides system change, accountability, education, and feedback are important to achieve and sustain hand hygiene compliance.
